# Assessing patient risk, benefit, and outcomes in drug development: A decade of ramucirumab clinical trials

**DOI:** 10.1002/cam4.7130

**Published:** 2024-05-02

**Authors:** Adam Khan, Hassan Khan, Griffin K. Hughes, Chase Ladd, Ryan McIntire, Brooke Gardner, Andriana M. Peña, Abigail Schoutko, Jordan Tuia, Kirstien Minley, Alyson Haslam, Vinay Prasad, Matt Vassar

**Affiliations:** ^1^ Office of Medical Student Research Oklahoma State University Center for Health Sciences Tulsa Oklahoma USA; ^2^ Department of Internal Medicine Oklahoma State University Center for Health Sciences Tulsa Oklahoma USA; ^3^ Department of Epidemiology and Biostatistics University of California San Francisco San Francisco California USA; ^4^ Department of Psychiatry and Behavioral Sciences Oklahoma State University Center for Health Sciences Tulsa Oklahoma USA

## Abstract

**Objective:**

This study aims to evaluate published clinical trials of ramucirumab to assess the risk/benefit profile and burden over time for patients.

**Background:**

The burden of oncologic drug development on patients paired with increasing clinical trial failure rates emphasizes the need for reform of drug development. Identifying and addressing patterns of excess burden can guide policy, ensure evidence‐based protections for trial participants, and improve medical decision‐making.

**Methods:**

On May 25, 2023 a literature search was performed on Pubmed/MEDLINE, Embase, Cochrane CENTRAL, and ClinicalTrials.gov for clinical trials using ramucirumab as monotherapy or in combination with other interventions for cancer treatment. Authors screened titles and abstracts for potential inclusion in a masked, duplicate fashion. Following data screening, data was extracted in a masked, duplicate fashion. Trials were classified as positive when meeting their primary endpoint and safety, negative or indeterminate.

**Results:**

Ramucirumab was initially approved for gastric cancer but has since been tested in 20 cancers outside of its FDA approved indications. In our analysis of ramucirumab trials, there were a total of 10,936 participants and 10,303 adverse events reported. Gains in overall survival and progression‐free survival for patients were 1.5 and 1.2 months, respectively. FDA‐approved indications have reported more positive outcomes in comparison to off‐label indications.

**Conclusion:**

We found that FDA‐approved indications for ramucirumab had better efficacy outcomes than non‐approved indications. However, a concerning number of adverse events were observed across all trials assessed. Participants in ramucirumab randomized controlled trials saw meager gains in overall survival when evaluated against a comparison group. Clinicians should carefully weigh the risks associated with ramucirumab therapy given its toxicity burden and poor survival gains.

## 1 INTRODUCTION

Gastric cancer remains a significant global health burden with high mortality and morbidity rates, particularly in advanced stages of the disease.^1^ Despite advancements in gastric cancer treatment there remains a need for new therapeutic approaches for disease management. Ramucirumab, a human monoclonal antibody targeting vascular endothelial growth factor receptor 2 (VEGFR2) emerged as a potential treatment option. Ramucirumab exerts its effects by selectively blocking the VEGF signaling pathway to inhibit angiogenesis, promoting antitumor effects.^2^ However, producing interventions for consumer use is complex—with analyses of drug development documenting increasing costs, delays in translation, and research waste.^3^, ^4^, ^5^ Moreover, patients must assess the risk, benefits, and barriers of trial participation.^6^ Patients participating in cancer clinical trials describe benefits including hope for a cure—while also detailing the burdens of adverse drug effects and out‐of‐pocket expenses.^7^, ^8^, ^9^ With less than 1 in 20 adult cancer patients enrolling in clinical trials, it remains relevant for all stakeholders to examine the total patient burden of trial participants.^6^

The high price of oncologic drug development coupled with high clinical trial failure rates have emphasized the need for initiatives to improve the pace of drug development.^10^ Yet, despite the push for accelerated drug development—it remains unknown what areas pose the most burden to patients. Recent studies have demonstrated the need for researching total patient burden throughout a drug's cumulative life cycle. Carlisle et al. report that following the initial FDA approval of imatinib, subsequent trials evaluating new drug combinations and indications were significantly less likely to attain FDA approval.^11^ Additionally, Carlisle et al. found worsening risk/benefit ratios as sunitinib clinical trials matured—with numerous negative, replicative trials.^12^ The risk/benefit variation throughout drug development raises ethical concerns for patient safety and research waste.

Identifying patterns of excess burden in drug development programs can improve research decision‐making, guide policy, and ensure evidence‐based human protections for trial participants.^12^ Given the ethical obligation to minimize risk and the need for novel cancer therapies–it is imperative to evaluate drug programs in their entirety and maximize the efficiency of clinical translation. Previous studies have explored the impact of ramucirumab on patient survival in Phase II/III trials but presently, no study has analyzed the risk/benefit profile for ramucirumab used in the treatment of advanced lung, gastric, liver, and colorectal cancers in Phase I–III trials.^13^, ^14^ Thus, our primary objective was to quantify the total risk/benefit profile for ramucirumab clinical trials and characterize areas of excess burden.

## 2 METHODS

### 2.1 Study design/openscience

We did a cross‐sectional study exploring clinical trials of ramucirumab (trade names *Cyramza*® or *Monoclonal Antibody HGS‐ETR2*®, Eli Lilly and Company) for their risk/benefit profiles throughout their development and applications to indications beyond initial approval. To improve rigor and reproducibility, as well as promote open science, we uploaded a protocol a priori to the investigation. Following study completion, we uploaded raw data, statistical analysis scripts, and extraction forms to Open Science Framework (OSF)—a free‐to‐upload data repository.^15^ Our data are accessible on OSF throughout the repository's lifecycle, or alternatively, it can be obtained by making a request. A red strikethrough and clarifying red text on OSF represented protocol amendments and erratum.

### 2.2 Research questions, definitions, and hypothesis

Given that clinical trials are costly and potentially harmful for patients, we aimed to evaluate the risks and benefits compared to the efficacy of ramucirumab in clinical trials. Our objective was to evaluate if the combined risk profiles represented an overall excessive risk to patients. A clinical trial *profile* was defined as the overall risk and benefit encountered by participants during a single trial as measured by selected tools mentioned in the *Data Extraction* section. A drug's *portfolio* was defined as the complete compilation of trial profiles for a given intervention. We hypothesized that the inclusion of off‐label indications in the expansion of clinical trials for ramucirumab would lead to a higher occurrence of negative trials with elevated patient risk, resulting in an overall negative drug portfolio.

### 2.3 Training

Author VP, an experienced clinical oncologist and expert in evidence‐based medicine, provided training to all authors on clinical trial design, reporting, and outcomes. Authors were educated on both the Common Terminology Criteria for Adverse Events (CTCAE)^16^ and Response Evaluation Criteria in Solid Tumors (RECIST).^17^ Screening authors were trained to use Rayyan (https://www.rayyan.ai/)^17^: an online tool to screen large samples of literature. A pilot‐tested Google extraction form was used for data extraction. Authors extracted and reviewed five studies from the sample that met the inclusion criteria. This preliminary step allowed the authors to train in the extraction process and familiarize themselves with the methodology before proceeding in data extraction.

### 2.4 Literature search

We performed a literature search on May 25, 2023 of PubMed/MEDLINE, Embase, Cochrane CENTRAL, and ClinicalTrials.gov for clinical trials using ramucirumab as monotherapy or in combination with other interventions for cancer treatment. Our search strings were standardized across these databases using the PolyGlot Search Translator (https://sr‐accelerator.com/#/polyglot) developed by the Institute for Evidence Based Healthcare and Bond University.^18^ Our search strings, including date of search and initial returns, were uploaded to OSF. They can be accessed as supplementary data in the final manuscript submission.

### 2.5 Selection process

We uploaded search returns into Rayyan for literature screening. Two authors (AK and HK) screened titles and abstracts for potential inclusion in a masked duplicate fashion. After screening was complete, author CL was available to resolve any discrepancies. We recorded reasons for exclusion during the screening process. This allowed us to create a flowchart for study exclusion.

### 2.6 Inclusion and exclusion criteria

The inclusion criteria we used in the screening process evaluated clinical trials that assessed: (1) adult and human subjects, (2) the efficacy of ramucirumab as a monotherapy or in combination with other agents to treat oncological malignancies, (3) the benefit of ramucirumab using radiographically derived response criteria for solid tumors (e.g., RECIST, mRECIST) and laboratory‐derived response criteria for nonsolid tumors, and (4) journals published in English. Non‐oncological studies, studies on nonsolid tumors, studies on biosimilars, pharmacology studies on healthy participants, and studies of exclusively pediatric populations were excluded. Studies that represented article types that are not clinical trials and non‐manuscript publication types including: secondary reports, interim results, clinical trial updates and follow‐ups, preclinical studies, literature reviews, systematic reviews, meta‐analyses, human tissue studies, laboratory studies, case reports, letters to the editor, editorials, opinion pieces, conference abstracts, corrections, or redactions were excluded.

### 2.7 Data extraction

Following literature screening, data extraction was performed on the clinical trial sample. Data extraction was done in a masked, duplicate fashion by two authors (AK and HK) with a third author (CL) available to resolve discrepancies. Authors extracted the following variables: published trial title, PubMed ID, clinical trial registry number, country of first author's affiliation, date of publication, metastatic or nonmetastatic stage, number of male participants, number of female participants, single‐center or multicenter, indication(s) of the trial, number of participants, mean or median age of participants, randomization ratio, study sponsor including conflicts of interest statements and funding, whether the trial was controlled, if the trial assessed monotherapy or combination therapies, phase of the trial, and blinding of trial participants.

For risk and benefit outcomes the following variables were extracted for treatment arms: the name of the arm, median progression‐free survival (PFS) in months, median overall survival (OS) in months, adverse events grade, partial response, complete response, objective response rate (ORR) as defined in the RECIST criteria, number of adverse events (Grades 3–5) as defined in the CTCAE criteria, and if the trial was positive, negative, or indeterminate. Using a prespecified indication, outcome measurements and adverse events encompassing all trial participants were extracted. A trial was classified as positive if it successfully achieved its prespecified endpoints while using a regimen that was well‐tolerated. On the other hand, a trial was categorized as negative if it failed to meet its prespecified endpoints or if it utilized a regimen that was not well‐tolerated. An indeterminate classification was assigned to trials that did not prespecify endpoints and utilized a regimen that was well‐tolerated. The tolerability of a regimen was determined by trial authors.^12^

Several design decisions were implemented concerning the specific characteristics of the trial. If multiple phases were reported in a trial, the higher phase was extracted. Exclusively partial responses were assumed when complete and partial were not specified. Further, when both confirmed and unconfirmed responses were reported, only the confirmed data was extracted. In dose‐escalation and dose expansion trials all arms were pooled for analysis. Mutations have been reported by their mutation classification. In crossover studies, all pertinent data were extracted prior to crossover to ensure accuracy. If a trial enrolled subjects in more than one indication then it was reported as “multiple indications.” A table detailing such trials was included in the supplementary data. Objective response rate was calculated for evaluable patients; however, if the trialist did not specify then all patients from the trial were included in the calculation.

### 2.8 Statistical analysis

A descriptive statistics in R (version 4.2.1) and RStudio was conducted.

### 2.9 Ethical oversight

Our protocol was reviewed by The Oklahoma State University Center for Health Sciences and it was determined that this research qualifies as nonhuman subjects research as defined in regulation 45 CFR 46.102(d) and (f).

## 3 RESULTS

### 3.1 General characteristics

**FIGURE 1:**
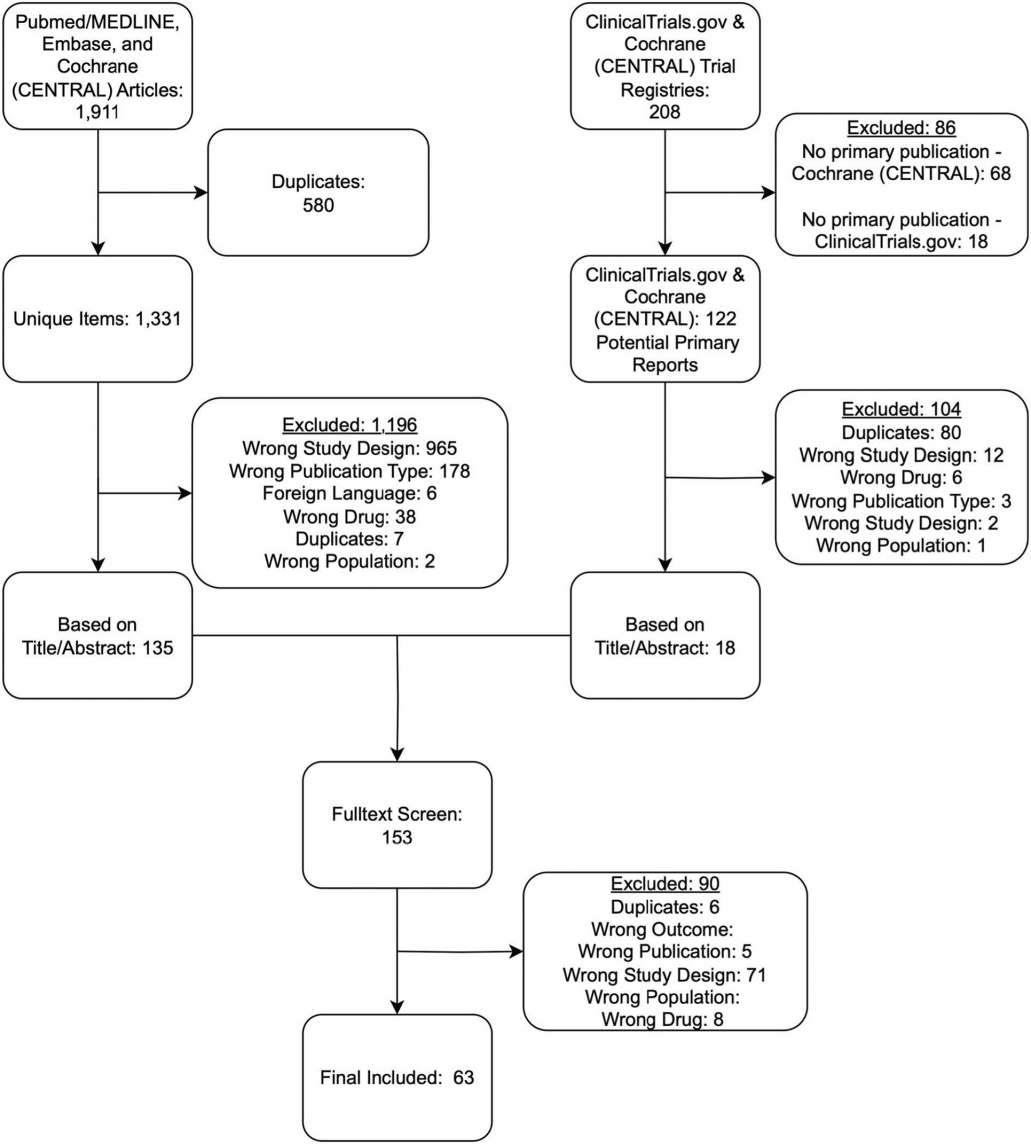
Flow diagram for study inclusion.

Our initial search identified 1911 studies for inclusion from bibliographic databases. The search on ClinicalTrials.gov found 208 additional trials for inclusion. After deduplication, abstracts and titles were screened, with 153 publications available for full‐text screening. Following full‐text screening 90 trials were further excluded, yielding a final sample of 63 publications (Figure 1).

Ramucirumab was tested in 24 indications across 63 clinical trials, including its four FDA‐approved indications (advanced hepatocellular carcinoma, gastric cancer, non‐small cell lung cancer, and metastatic colorectal cancer) in addition to off‐label use. The most common indications for ramucirumab were non‐small cell lung cancer (10; 15.9%), gastric cancer (9; 14.3%), and gastric or gastroesophageal junction cancer (9; 14.3%). A total of 10,936 patients were included within our dataset, with 56% being male and 42% being female (Table 1). Of the 63 trials, 26 (41.3%) were randomized and 37 (58.7%) were nonrandomized. Fifty‐one (81%) were combination trials while 12 (19%) evaluated ramucirumab as monotherapy. Thirty‐six studies (57.1%) had positive trial results, 23 (36.5%) had negative, and 4 (6.4%) had indeterminate (Table 2).

**TABLE 1:** Characteristics of included trials.

Characteristic	Overall (*N* = 63)	Combination (*N* = 51)	Monotherapy (*N* = 12)
Phase			
Phase I	20 (31.8%)	17 (33.3%)	3 (25.0%)
Phase II	31 (49.2%)	25 (49.0%)	6 (50.0%)
Phase III	12 (19.1%)	9 (17.7%)	3 (25.0%)
Stage			
Metastatic	62 (98.4%)	50 (98.0%)	12 (100.0%)
Nonmetastatic	1 (1.6%)	1 (2.0%)	0 (0.0%)
Response criteria used			
RECIST	62 (98.4%)	50 (98.0%)	12 (100.0%)
Other	1 (1.6%)	1 (2.0%)	0 (0.0%)
Results			
Positive	37 (58.7%)	32 (62.8%)	4 (33.3%)
Negative	23 (36.5%)	16 (31.4%)	7 (58.3%)
Indeterminate	3 (4.8%)	3 (5.9%)	1 (8.3%)
Randomization			
Non‐randomized	37 (58.7%)	29 (56.9%)	8 (66.7%)
Randomized	26 (41.3%)	22 (43.1%)	4 (33.3%)
Randomization ratio			
1:1	17 (68.0%)	16 (76.2%)	1 (25.0%)
2:1	5 (20.0%)	3 (14.3%)	2 (50.0%)
1:1:1	2 (8.0%)	2 (9.5%)	0 (0.0%)
2:1:2:1	1 (4.0%)	0 (0.0%)	1 (25.0%)
Unknown	38	30	8
Blinding			
Non‐blinded	45 (71.4%)	38 (74.5%)	7 (58.3%)
Double	18 (28.6%)	13 (25.5%)	5 (41.7%)
Number of centers			
Multicenter	50 (79.4%)	41 (80.4%)	9 (75.0%)
Single‐center	7 (11.1%)	6 (11.8%)	1 (8.3%)
Sponsor/funding			
Industry	48 (76.2%)	38 (74.5%)	10 (83.3%)
Industry, government	1 (1.6%)	0 (0.0%)	1 (8.3%)
Industry, government, non‐industry	2 (3.2%)	2 (3.9%)	0 (0.0%)
Industry, non‐industry	3 (4.8%)	3 (5.9%)	0 (0.0%)
Non‐industry	2 (3.2%)	1 (2.0%)	1 (8.3%)
Not funded	1 (1.6%)	1 (2.0%)	0 (0.0%)
Not stated	6 (9.5%)	6 (11.8%)	0 (0.0%)
Conflict of interest/disclosure statement			
Reports conflicts of interest	58 (92.1%)	46 (90.2%)	12 (100.0%)
Reports no conflicts of interest	5 (7.9%)	5 (9.8%)	0 (0.0%)
Country			
United States	25 (39.7%)	17 (33.3%)	8 (66.7%)
Japan	20 (31.8%)	19 (37.3%)	1 (8.3%)
United Kingdom	3 (4.8%)	2 (3.9%)	1 (8.3%)
Spain	3 (4.8%)	3 (5.9%)	0 (0.0%)
Germany	3 (4.8%)	3 (5.9%)	0 (0.0%)
Canada	3 (4.8%)	2 (3.9%)	1 (8.3%)
China	2 (3.2%)	1 (2.0%)	1 (8.3%)
Italy	1 (1.6%)	1 (2.0%)	0 (0.0%)
Republic of Korea	1 (1.6%)	1 (2.0%)	0 (0.0%)
South Korea	1 (1.6%)	1 (2.0%)	0 (0.0%)
Taiwan	1 (1.6%)	1 (2.0%)	0 (0.0%)
Journal			
Annals of Oncology	3 (4.8%)	2 (3.9%)	1 (8.3%)
Anticancer Research	2 (3.2%)	2 (3.9%)	0 (0.0%)
British Journal of Cancer	1 (1.6%)	1 (2.0%)	0 (0.0%)
Cancer	2 (3.2%)	1 (2.0%)	1 (8.3%)
Cancer Chemotherapy and Pharmacology	3 (4.8%)	3 (5.9%)	0 (0.0%)
Cancers	1 (1.6%)	1 (2.0%)	0 (0.0%)
Clinical Breast Cancer	1 (1.6%)	1 (2.0%)	0 (0.0%)
Clinical Cancer Research	5 (7.9%)	3 (5.9%)	2 (16.7%)
eClinicalMedicine	1 (1.6%)	1 (2.0%)	0 (0.0%)
European Journal of Cancer	7 (11.1%)	7 (13.7%)	0 (0.0%)
Gastric Cancer	1 (1.6%)	0 (0.0%)	1 (8.3%)
Genome Medicine	1 (1.6%)	1 (2.0%)	0 (0.0%)
Gynecologic Oncology	1 (1.6%)	0 (0.0%)	1 (8.3%)
International Journal of Cancer	1 (1.6%)	1 (2.0%)	0 (0.0%)
International Journal of Clinical Oncology	1 (1.6%)	1 (2.0%)	0 (0.0%)
Investigational New Drugs	1 (1.6%)	1 (2.0%)	0 (0.0%)
JAMA Network Open	1 (1.6%)	1 (2.0%)	0 (0.0%)
Japanese Journal of Clinical Oncology	1 (1.6%)	1 (2.0%)	0 (0.0%)
Journal of Clinical Oncology	3 (4.8%)	2 (3.9%)	1 (8.3%)
Journal of Thoracic Oncology	1 (1.6%)	1 (2.0%)	0 (0.0%)
Lung Cancer	3 (4.8%)	3 (5.9%)	0 (0.0%)
Supportive Care in Cancer	1 (1.6%)	1 (2.0%)	0 (0.0%)
Targeted Oncology	1 (1.6%)	1 (2.0%)	0 (0.0%)
The Lancet	2 (3.2%)	1 (2.0%)	1 (8.3%)
The Lancet Oncology	9 (14.3%)	6 (11.8%)	3 (25.0%)
The Oncologist	9 (14.3%)	8 (15.7%)	1 (8.3%)

**TABLE 2:** Overall trial characteristics and outcomes by indication.

Solid tumor indication	No. of trials	No. of randomized trials *n* (%)	No. of participants	No. of males	No. of females	No. of grade 3–5 events	Median age	Median PFS (months)	Median OS (months)	Median partial response rate (%)	Median complete response rate (%)	Median ORR^a^ (%)
Non‐small cell lung cancer	10	5 (50)	2349	1339	908	2985	64.45	5.2	14.6	34.10	0.20	34.10
Gastric cancer	9	1 (11.1)	544	372	166	517	66	5.3	12.9	35.50	0.00	35.50
Gastric or gastroesophageal junction cancer	9	4 (44.4)	2117	1444	635	2387	61	3.8	9.3	23.2	0.9	25.0
Metastatic colorectal cancer	6	2 (33.3)	1337	745	567	1267	62	4.9	11.7	12.2	0.0	13.4
Advanced hepatocellular carcinoma	5	3 (60)	1012	837	174	566	62.5	2.8	8.5	3.7	0.0	3.7
Metastatic breast cancer	4	3 (75)	1445	1	1441	1098	55.1	4.4	15.5	30.5	0.9	31.8
Biliary tract cancer	3	1 (33.3)	395	179	216	316	62	6.5	10.5	16.7	0.0	19.6
Multiple indications	7	0 (0)	409	257	151	141	60.6	2.9	11	6.7	0.0	6.7
Epithelial ovarian, fallopian tube or primary peritoneal carcinoma	1	0 (0)	73	0	73	30	62	3.5	11.1	5.0	0.0	5.0
Esophagogastric adenocarcinoma	1	1 (100)	180	106	46	228	60	—^b^	45.5	—	—	—
Lung adenocarcinoma	1	0 (0)	6	4	2	1	67	9.2	—	83.3	0.0	83.3
Malignant pleural mesothelioma	1	1 (100)	165	119	42	59	69	4.9	10.7	8.1	0.0	8.1
Metastatic melanoma	1	1 (100)	106	75	27	61	62.25	2.2	9.9	10.7	0.0	10.7
Metastatic prostate cancer	1	1 (100)	132	132	0	161	66.5	5.4	11.9	23.4	0.0	23.4
Pancreatic cancer	1	1 (100)	86	39	43	19	62.36	6.2	10	18.5	1.6	20.1
Renal cell carcinoma	1	0 (0)	40	31	8	8	59	7.1	24.8	5.1	2.6	7.7
Small cell lung cancer	1	0 (0)	10	8	2	10	63	7.2	22.4	100.0	0.0	100.0
Urothelial carcinoma	1	1 (100)	530	428	102	449	65.5	3.5	8.7	17.6	2.3	19.9
Totals/medians	63	26 (42.6)	10936	6116	4603	10303	62.305	4.4	11.4	22.7	0.9	23.6

^a^ Response rate median calculations for those indications using RECIST or mRECIST only.

^b^ Did not measure outcome, failed to report outcomes, or failed to report outcome measure for all enrolled patients.

### 3.2 Endpoints

The most common endpoints in our sample were safety and PFS. Progression‐free survival was the primary endpoint in 24 (of 63; 38%) studies with 5 (of 24, 21%) trials reporting positive outcomes. Of the 63 trials in our study, 15 (23.8%) did not report an OS and 10 (15.9%) did not report a PFS. The median PFS and OS were 4.4 and 11.4 months, respectively. Esophagogastric adenocarcinoma had the highest OS (45.5 months), and urothelial carcinoma had the lowest OS (8.65 months). Lung adenocarcinoma had the highest PFS (9.2 months) and metastatic melanoma had the lowest PFS (2.2 months). Combination therapy had a higher PFS (4.5 months) and OS (11.7 months) than monotherapy (PFS: 3 months, OS: 9.4 months) (Table 1, 3, 4).

**TABLE 3:** Monotherapy trial characteristics and outcomes by indication.

Solid tumor indication	No. of trials	No. of randomized trials, *n* (%)	No. of participants	No. of males	No. of females	No. of grade 3–5 events	Median age	Median PFS (months)	Median OS (months)	Median partial response rate (%)	Median complete response rate (%)	Median ORR^a^ (%)
Advanced hepatocellular carcinoma	3	2 (66.7)	900	745	154	468	64	2.8	8.5	4.6	0.0	4.6
Biliary tract cancer	2	1 (50)	369	171	198	309	60.9	6.6	11.8	23.7	0.1	25.4
Epithelial ovarian, fallopian tube or primary peritoneal carcinoma	1	0 (0)	73	0	73	30	62	3.5	11.1	5.0	0.0	5.0
Gastric or gastroesophageal junction cancer	2	1 (50)	391	269	122	184	60	1.5	5.2	2.6	0.0	2.6
Multiple indications	3	0 (0)	90	53	37	50	57	—^b^	—	0.0	0.0	0.0
Renal cell carcinoma	1	0 (0)	40	31	8	8	59	7.1	24.8	5.1	2.6	7.7
Totals/medians	12	4 (33.3)	1863	1269	592	1049	62	3	9.4	7.40	0.40	7.80

^a^ Response rate median calculations for those indications using RECIST or mRECIST only.

^b^ Did not measure outcome, failed to report outcomes, or failed to report outcome measure for all enrolled patients.

**TABLE 4:** Combination therapy trial characteristics and outcomes by indication.

Solid tumor indication	No. of trials	No. of randomized trials, *n* (%)	No. of participants	No. of males	No. of females	No. of grade 3–5 events	Median age	Median PFS (months)	Median OS (months)	Median partial response rate (%)	Median complete response rate (%)	Median ORR^a^ (%)
Advanced hepatocellular carcinoma	2	1 (50)	112	92	20	98	55.6	2.2	7.7	2.9	0.0	2.9
Biliary tract cancer	1	0 (0)	26	8	18	7	63.0	1.6	6.4	3.8	0.0	3.8
Esophagogastric adenocarcinoma	1	1 (100)	180	106	46	228	60.0	—^b^	45.5	—	—	—
Gastric cancer	9	1 (11.1)	544	372	166	517	66.0	5.3	12.9	35.5	0.0	35.5
Gastric or gastroesophageal junction cancer	7	4 (57.1)	1726	1175	513	2203	61.0	4.4	10.7	27.3	1.6	27.9
Lung adenocarcinoma	1	0 (0)	6	4	2	1	67.0	9.2	—	83.3	0.0	83.3
Malignant pleural mesothelioma	1	1 (100)	165	119	42	59	69.0	4.9	10.7	8.1	0.0	8.1
Metastatic breast cancer	4	3 (75)	1445	1	1441	1098	55.1	4.4	15.5	30.5	0.9	31.8
Metastatic colorectal cancer	6	2 (33.3)	1337	745	567	1267	62.0	4.9	11.7	12.2	0.0	13.4
Metastatic melanoma	1	1 (100)	106	75	27	61	62.3	2.2	9.9	10.7	0.0	10.7
Metastatic prostate cancer	1	1 (100)	132	132	0	161	66.5	5.4	11.9	23.4	0.0	23.4
Multiple indications	4	0 (0)	319	204	114	91	61.3	2.9	11	7.6	0.0	7.6
Non‐small cell lung cancer	10		2349	1339	908	2985	64.5	5.2	14.6	34.1	0.2	34.1
Pancreatic cancer	1	1 (100)	86	39	43	19	62.4	6.15	10	18.5	1.6	20.1
Small cell lung cancer	1	0 (0)	10	8	2	10	63.0	7.2	22.4	100.0	0.0	100
Urothelial carcinoma	1	1 (100)	530	428	102	449	65.6	3.5	8.7	17.6	2.3	19.9
Totals/Medians	51	22 (43.1)	9073	4847	4011	9254	62.0	4.5	11.7	27.10	1.10	28.2

^a^ Response rate median calculations for those indications using RECIST or mRECIST only.

^b^ Did not measure outcome, failed to report outcomes, or failed to report outcome measure for all enrolled patients.

### 3.3 Delta PFS and OS

Twenty‐three randomized controlled trials were analyzed for ΔPFS and ΔOS. The median change in the randomized controlled trials for OS was 1.5 months and PFS was 1.2 months between the ramucirumab treatment arm and the comparison arm. The highest ΔPFS (7.0 months) was in a trial for non‐small cell lung cancer that led to FDA approval. FDA‐approved indications reported statistically significant ΔPFS values when evaluated against a comparison group. Pancreatic cancer, an off‐label indication, had the lowest ΔPFS (−1.1 months) and ΔOS (0.6 months) (Table 5).

**TABLE 5:** ∆ PFS and OS for randomized control trials.

Trial	Phase	Result	Date	Indication	Ramucirumab group	Comparison group	∆PFS^a^	PFS significance	∆OS^a^	OS Significance
NCT02435433	3	Positive	06‐10‐2022	Advanced hepatocellular carcinoma	Ramucirumab + BSC	Placebo + BSC	1.5	NA	2.9	NA
NCT02661971	3	Negative	23‐03‐2023	Esophagogastric adenocarcinoma	FLOT + RAM	FLOT	NA	NA	1	Not significant [*p* = 0.749]
NCT02539225	2	Negative	02‐08‐2019	Gastric cancer	RAM+SOX	PBO+SOX	–0.4	Not significant [*p* = 0.7]	0.4	Not significant [*p* = 0.55]
NCT01170663	3	Positive	17‐09‐2014	Gastric or gastroesophageal junction cancer	Ramucirumab plus paclitaxel	Placebo plus paclitaxel	1.5	Significant [*p* < 0.0001]	2.2	Significant [*p* = 0.017]
NCT01246960	2	Negative	20‐10‐2016	Gastric or gastroesophageal junction cancer	RAM + mFOLFOX6	PBO + mFOLFOX6	–0.3	Not significant [*p* = 0.886]	0.2	Not significant [*p* = 0.712]
NCT02314117	3	Positive	01‐02‐2019	Gastric or gastroesophageal junction cancer	Ramucirumab plus fluoropyrimidine and cisplatin	Placebo plus fluoropyrimidine and cisplatin	0.3	Significant [*p* = 0.0106]	0.5	Not significant [*p* = 0.6757]
NCT03081143	2	Negative	21‐02‐2022	Gastric or gastroesophageal junction cancer	Paclitaxel + ramucirumab	FOLFIRI + ramucirumab	NA	NA	NA	NA
NCT03560973	2	Positive	06‐09‐2021	Malignant pleural mesothelioma	Gemcitabine plus ramucirumab	Gemcitabine plus placebo	3.1	Significant [*p* = 0.082]	6.3	Significant [*p* = 0.028]
NCT00703326	3	Negative	02‐09‐2014	Metastatic breast cancer	RAM plus DOC	PBO Plus DOC	1.3	Significant [*p* = 0.008]	0.1	Significant [*p* = 0.915]
NCT01427933	2	Negative	29‐07‐2016	Metastatic breast cancer	Ramucirumab with eribulin	Eribulin	0.3	Not significant [*p* = 0.35]	2	Not significant [*p* = 0.68]
NCT01183780	3	Positive	12‐04‐2015	Metastatic colorectal cancer	Ramucirumab + folfiri	Placebo + folfiri	1.2	Significant [*p* < 0.0005]	1.6	Significant [*p* = 0.0219
NCT00533702	2	Indeterminate	12‐06‐2014	Metastatic melanoma	RAM	RAM + DTIC	NA	NA	NA	NA
NCT00683475	2	Positive	13‐06‐2015	Metastatic prostate cancer	Ramucirumab + M + P	Cixutumumab + M + P	2.6	NA	2.2	NA
NCT01168973	3	Positive	02‐06‐2014	Non‐small cell lung cancer	Ramucirumab plus docetaxel	Placebo plus docetaxel	1.5	Significant [*p* < 0.0001]	1.4	Significant [*p* = 0.023]
NCT01160744	2	Negative	06‐11‐2014	Non‐small cell lung cancer	RAM + PEM + Cb/Cis	PEM + Cb/Cis	1.6	Not significant [*p* = 0.1318]	3.5	Not significant [*p* = 0.8916]
NCT01703091	2	Positive	18‐07‐2016	Non‐small cell lung cancer	Ramucirumab‐docetaxel	Placebo‐docetaxel	1	NA	0.5	NA
NCT02411448	3	Positive	04‐10‐2019	Non‐small cell lung cancer	Ramucirumab plus erlotinib	Placebo plus erlotinib	7	Significant [*p* = 0.0060]]	NA	NA
NCT03971474	2	Positive	03‐06‐2022	Non‐small cell lung cancer	Ramucirumab and pembrolizumab	Standard of care	–0.7	Not significant [*p* = 0.25]	2.9	Significant [*p* = 0.05]
NCT02581215	2	Negative	11‐03‐2023	Pancreatic cancer	mFOLFIRINOX plus ramucirumab	mFOLFIRINOX plus placebo	–1.1	NA	0.6	NA
NCT02426125	3	Negative	18‐11‐2019	Urothelial carcinoma	Ramucirumab plus docetaxel	Placebo plus docetaxel	1.3	Significant [*p* = 0.0002	1.5	Not significant [*p* = 0.25]
NCT01140347	3	Negative	18‐06‐2015	Advanced hepatocellular carcinoma	Ramucirumab	Placebo + BSC	0.7	Significant [*p* < 0.0001]	1.6	Not significant [*p* = 0.14]
NCT02435433	3	Positive	18‐01‐2019	Advanced hepatocellular carcinoma	Ramucirumab	Placebo group + BSC	1.2	Significant [*p* < 0.0001]	1.2	Significant [*p* = 0.0199]
NCT00917384	3	Positive	03‐10‐2013	Gastric or gastroesophageal junction cancer	Ramucirumab	Placebo + BSC	0.8	Significant [*p* < 0.0001]	1.4	Significant [*p* = 0.047]

^a^ Months.

### 3.4 Risk assessment

**FIGURE 2:**
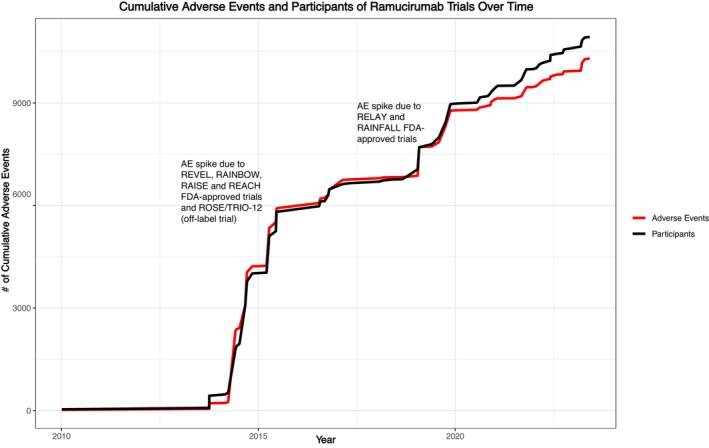
Cumulative adverse events and participants of ramucirumab trials over time.

In our analysis of ramucirumab trials, there were a total of 10,936 participants and 10,303 adverse events reported. A spike in adverse events observed in 2014–2015 occurred after publication of Phase III trials RAINBOW, REVEL, ROSE/TRIO‐12, RAISE, and REACH, which enrolled a combined 5342 patients and had 4927 cumulative Grade 3–5 adverse events. An additional peak was seen in 2019 following Phase III trials RAINFALL and RELAY, which enrolled 1094 patients and had 1285 cumulative Grade 3–5 adverse events. Adverse events increased with participant enrollment until 2020. However, since then, this ratio has demonstrated a decline, as seen in Figure 2.

**FIGURE 3:**
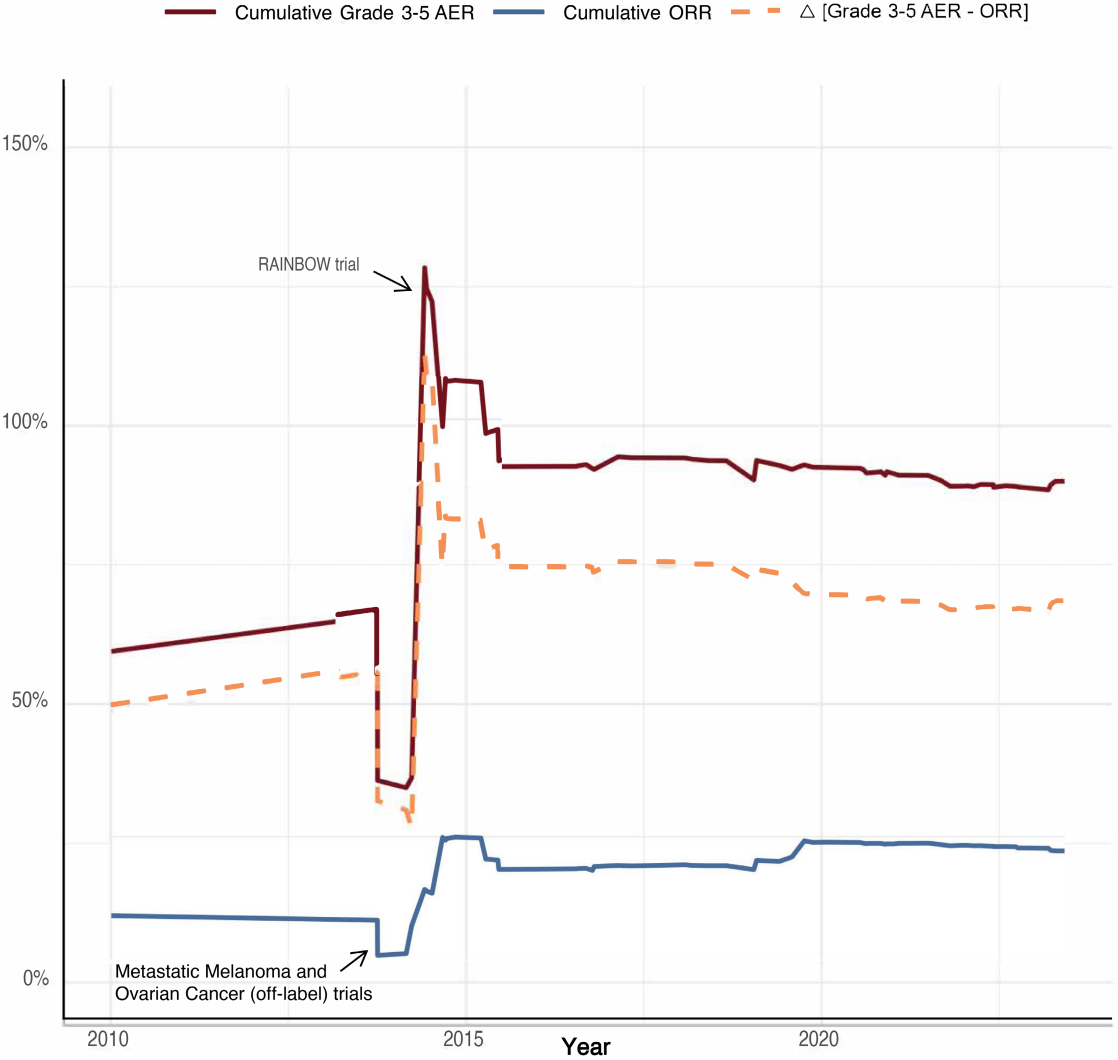
Cumulative adverse event rate and cumulative ORR.

As shown in Figure 3, the cumulative adverse event rate (AER) was over 100%, illustrating more adverse events than patients, following the RAINBOW trial in 2014. Following ramucirumab testing for NSCLC, HCC, and gastric cancer from 2010 to 2013, indication use began to be expanded. A metastatic melanoma and ovarian cancer trial conducted in 2014, which had response rates of 10.8% and 5%, brought down cumulative ORR to the lowest values in ramucirumab's trial history. The same year, the highest cumulative ORR was observed, likely due to the ROSE/TRIO‐12 and RAINBOW trials which reported ORR values of 42.4% and 22%, respectively. The AER, in relation to the cumulative ORR, remains elevated compared to pre‐initial FDA‐approval levels, suggesting an increase in overall risk.

### 3.5 Accumulating evidence and research organization (AERO) diagram

**FIGURE 4:**
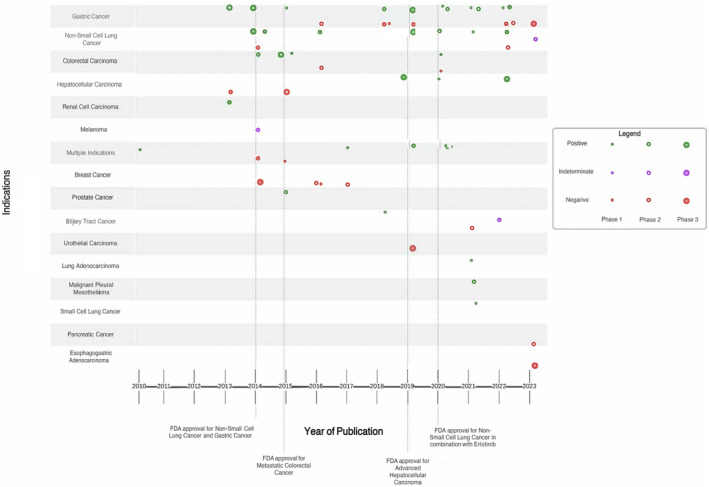
AERO diagram representing trial progression.

Figure 4 provides a visual representation of the ramucirumab clinical trial portfolio, capturing the progression of its development through different indications and phases. Ramucirumab has had a total of six FDA approvals for four indications with the first approval occurring in 2014 and the most recent in 2020. Advanced gastric cancer and non‐small cell lung cancer were approved in 2014, metastatic colorectal cancer in 2015, and advanced hepatocellular carcinoma in 2019.

**FIGURE 5:**
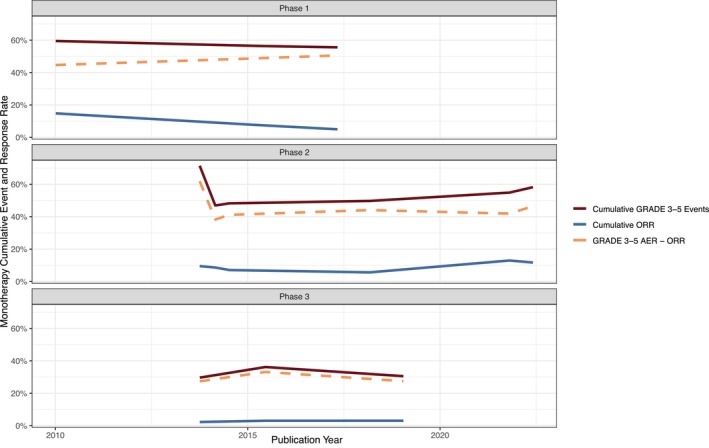
Cumulative Grade 3–5 events and cumulative ORR in Phases 1–3 of combination trials.

**FIGURE 6:**
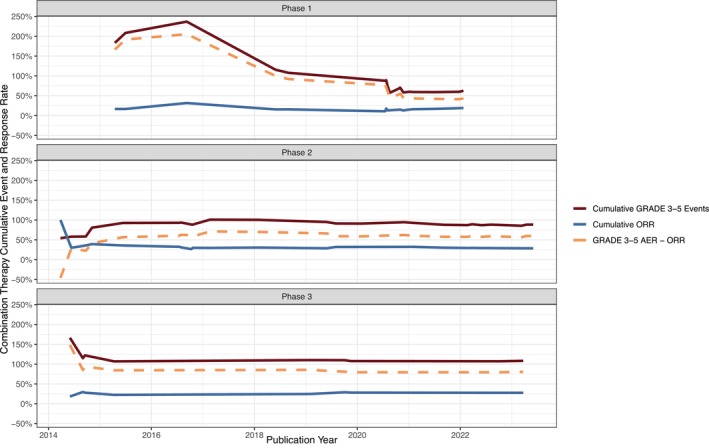
Cumulative Grade 3–5 events and cumulative ORR in Phases 1–3 of monotherapy trials.

After 2014 there was an expansion into 21off‐label trials, and it is notable that 47% (10 out of 21) of these trials yielded negative results, with none of them receiving FDA approval. Overall, the percentage of positive trials for FDA‐approved indications was 21.4% greater than the percentage of non‐FDA approved indications, suggesting superior efficacy outcomes for on‐label trials. There were 51 Phase I and II studies in our sample, and 12 Phase III studies, indicating limited progression past Phase II. Additionally, Figures 5 and 6 provide a visual representation of cumulative Grade 3–5 events and cumulative ORR in Phases 1–3 of both combination and monotherapy trials. Ultimately, our results indicate that, for clinical trials of both FDA‐approved and non‐FDA approved indications of ramucirumab, there is an increase in risk for patients over time. However, FDA‐approved indication trials of ramucirumab are associated with prolonged survival when compared to off‐label, or novel, indication trials.

## 4 DISCUSSION

Ramucirumab is a next in class monoclonal antibody targeting the VEGFR‐2.^19^ It initially received FDA approval in 2014 for the treatment of metastatic gastric cancer in the second line setting and most recently received FDA approval in 2020 for the first‐line treatment of metastatic EGFR‐mutated non‐small cell lung cancer. Since then, the drug has gained regulatory authorization in other metastatic tumor types, specifically non‐small cell lung cancer, hepatocellular carcinoma, and colorectal cancer. Out of the 24 total indications in our study, 4 were FDA approved. We sought to put the regulatory authorization in the broader context of the clinical trials portfolio of this drug.

In our study, we performed an in‐depth evaluation of clinical trials focusing on the use of ramucirumab to treat solid tumors published within the last 13 years. Our results showed a varied response when investigating ramucirumab beyond its original FDA‐approved indications for gastric and gastroesophageal carcinoma. We also observed a significant increase in cumulative Grade 3–5 adverse events between the years 2014 and 2017, accompanied by a proportional decrease in ORR. This finding coincides with several studies exploring off‐label use of ramucirumab.

Patients treated with ramucirumab experienced a modest gain of 1.5 months in median OS. This result may indicate the limited effectiveness of ramucirumab in enhancing survival rates. Nonetheless, it is notable that the change in OS and PFS still demonstrate higher values for FDA‐approved indications of ramucirumab than off‐label indications. Effing and Gyawali, observed gains in median overall survival ranging from 1.2 to 2.2 months and determined these were marginal improvements in relation to the toxicity experienced by patients.^20^ These findings emphasize the importance of carefully evaluating the efficacy of ramucirumab by clinicians when making treatment decisions.

Additionally, FDA‐approved indications for ramucirumab exhibited higher ORR, PFS, and OS when compared to non‐approved indications. Grabowski et al. found benefits when ramucirumab was used as treatment for HCC, NSCLC, colorectal cancer, and gastric cancer.^21^ However, following ramucirumab's FDA approval in 2014, trialists began investigating its efficacy in various forms of cancer. Our results revealed a wide range of outcomes, with trials yielding both positive and negative effects. For example, multiple studies were conducted examining the efficacy in treatment for both biliary tract cancer and metastatic breast cancer. Unfortunately, these studies have produced predominantly negative responses with trialists acknowledging efficacy outcomes were insufficient for treatment.^22^, ^23^ Studies conducted by Carlisle et al. on imatinib and sunitinib concluded that indications beyond the scope of FDA approval were only tested after the strongest indications were thoroughly examined and these trials were associated with more negative results.^11^, ^12^ Our results align with these findings, as testing for indications without strong preclinical evidence, such as breast cancer and ovarian cancer, were associated with more negative trial results and occurred once FDA approval for ramucirumab's initial indications were thoroughly examined. As a VEGFR‐2 inhibitor, ramucirumab inhibits angiogenesis, an important mechanism for many tumors.^24^ Neutralizing tumor progression, and extending life, are important efficacy markers for cancer therapeutics.

Although our results indicate over a third, or 23 of 36, of trials were negative, nevertheless for certain indications, notably gastric cancer and NSCLC, ramucirumab was found to be associated with prolonged OS and PFS. Specifically, ramucirumab was associated with a higher PFS, OS, and ORR in combination therapy than monotherapy. Given that the majority of ramucirumab's FDA‐approvals are for its treatment as a combination therapy, this finding is to be anticipated. Ramucirumab has been found to enhance inhibitory effects in combination, allowing for more efficacious tumor containment.^25^, ^26^ Prior analyses demonstrate that ramucirumab used as a combination therapy for lung, gastric, and colorectal cancers has high efficacy, PFS, and OS.^27^, ^28^, ^29^ While clear benefits exist in regards to this treatment's efficacy, understanding the toxicity profile is important to take into consideration as well.

Further, our study revealed a notable risk period in the development of ramucirumab, with the years 2014–2017 exhibiting the highest cumulative Grade 3–5 adverse events and proportionally lowest cumulative ORR. Specifically, a significant contributor to this occurrence was a 2014 study that examined the efficacy of ramucirumab as a combination therapy for the treatment of gastric or gastroesophageal junction cancer. This study reported 72 total Grade 5 events in 647 patients. It is important to recognize that a case‐specific investigation is warranted before attributing the cause of these Grade 5 events directly to the toxicity of ramucirumab. Despite a low ORR of 16%, the trial was classified as positive as it met the prespecified primary endpoint of median OS even with a high AER. The authors concluded the toxicity was acceptable, as the reported incidences were within the range of previous large Phase 3 gastric cancer trials. Additionally, four clinical trials were conducted on ramucirumab for the treatment of metastatic breast cancer, an off‐label indication. Three of the four studies had negative results and were associated with 1098 Grade 3–5 adverse events. These findings align with previous research by Carlisle, et al. who observed a similar pattern in the development of two cancer drugs, imatinib and sunitinib, where efforts to apply these drugs to new indications led to repeated negative results and increased burden on trial participants.^11^, ^12^ The financial and toxicity burden associated with ramucirumab calls for future studies to focus on refining patient selection to maximize the benefit from this treatment.

Our data highlights the ethics of continuing off‐label trials in light of negative results. While there are circumstances where off‐label use is justifiable, such as in compassionate care cases and exhausted lines of treatment situations, the patient burden, research waste, and overall risk/benefit profile of a drug must be considered. Although clinical trials in oncology inherently involve risk for patients, researchers must continually ensure the potential benefits outweigh the harms. Our results indicate when two non‐FDA‐approved trials were conducted limited efficacy was observed. This presents an ethical dilemma where patients, in hopes of gaining access to treatments which could improve their health, are more willing to participate in riskier trials.^30^ These harms directly upset the risk/benefit analysis of a therapeutic, which may be additionally amplified when assessing newer drugs given shorter time frames of most trials may not capture the true toxicity profile.^31^ Providing patients with therapeutics that may improve their outcome should be the goal for clinical trials. Trialists should strive to uphold these standards and closely examine efficacy outcomes before proceeding to conduct additional studies on the same indication.

### 4.1 Strengths and limitations

Our study has several strengths. First, investigators received data extraction training and conducted the study in a masked, duplicate fashion in accordance with the Cochrane Collaboration Guidelines.^32^ Second, we used Rayyan, a reliable search platform for systematic reviews, to conduct the title and abstract screen.^33^ Lastly, we uploaded our extraction form, data sheet, and protocol to OSF, an open‐source repository that promotes transparency in research.^14^ Despite being methodologically rigorous our study is not without limitations. First, our initial systematic search may not have yielded all relevant publications. In addition, although we applied measures to minimize errors in data extraction, there is a possibility that mistakes persisted. Lastly, our study cannot be applied to other medications or fields of medicine as it is a cross‐sectional design.

## 5 CONCLUSION

We sought to quantify the risk/benefit profile of ramucirumab clinical trials and characterize areas of excess burden. Overall, FDA‐approved indications had better efficacy outcomes than non‐approved indications. However, a concerning number of adverse events were observed across all trials, even those that led to FDA approval. Participants in ramucirumab randomized controlled trials saw meager gains of only 1.5 months in overall survival when evaluated against a comparison group. Given the toxicity burden of ramucirumab, combined with poor survival gains, clinicians should weigh the potential benefits against the potential risks and adverse events. Future research and evaluations are essential to obtain a more thorough understanding of the optimal utilization and potential risks associated with ramucirumab treatment.

## AUTHOR CONTRIBUTIONS

**Adam Khan:** Conceptualization (equal); data curation (equal); formal analysis (equal); funding acquisition (equal); investigation (equal); methodology (equal); project administration (equal); resources (equal); software (equal); supervision (equal); validation (equal); visualization (equal); writing – original draft (equal); writing – review and editing (equal). **Hassan Khan:** Conceptualization (equal); data curation (equal); formal analysis (equal); funding acquisition (equal); investigation (equal); methodology (equal); project administration (equal); resources (equal); software (equal); supervision (equal); validation (equal); visualization (equal); writing – original draft (equal); writing – review and editing (equal). **Griffin Hughes:** Conceptualization (equal); data curation (equal); formal analysis (equal); funding acquisition (equal); investigation (equal); methodology (equal); project administration (equal); resources (equal); software (equal); supervision (equal); validation (equal); visualization (equal); writing – original draft (equal); writing – review and editing (equal). **Chase Ladd:** Conceptualization (equal); data curation (equal); formal analysis (equal); funding acquisition (equal); investigation (equal); methodology (equal); project administration (equal); resources (equal); software (equal); supervision (equal); validation (equal); visualization (equal); writing – original draft (equal); writing – review and editing (equal). **Ryan McIntire:** Conceptualization (equal); data curation (equal); formal analysis (equal); funding acquisition (equal); investigation (equal); methodology (equal); project administration (equal); resources (equal); software (equal); supervision (equal); validation (equal); visualization (equal); writing – original draft (equal); writing – review and editing (equal). **Brooke Gardner:** Conceptualization (equal); data curation (equal); formal analysis (equal); funding acquisition (equal); investigation (equal); methodology (equal); project administration (equal); resources (equal); software (equal); supervision (equal); validation (equal); visualization (equal); writing – original draft (equal); writing – review and editing (equal). **Andriana Peña:** Conceptualization (equal); data curation (equal); formal analysis (equal); funding acquisition (equal); investigation (equal); methodology (equal); project administration (equal); resources (equal); software (equal); supervision (equal); validation (equal); visualization (equal); writing – original draft (equal); writing – review and editing (equal). **Abigail Schoutko:** Conceptualization (equal); data curation (equal); formal analysis (equal); funding acquisition (equal); investigation (equal); methodology (equal); project administration (equal); resources (equal); software (equal); supervision (equal); validation (equal); visualization (equal); writing – original draft (equal); writing – review and editing (equal). **Jordan Tuia:** Conceptualization (equal); data curation (equal); formal analysis (equal); funding acquisition (equal); investigation (equal); methodology (equal); project administration (equal); resources (equal); software (equal); supervision (equal); validation (equal); visualization (equal); writing – original draft (equal); writing – review and editing (equal). **Kirstien Minley:** Conceptualization (equal); data curation (equal); formal analysis (equal); funding acquisition (equal); investigation (equal); methodology (equal); project administration (equal); resources (equal); software (equal); supervision (equal); validation (equal); visualization (equal); writing – original draft (equal); writing – review and editing (equal). **Alyson Haslam:** Conceptualization (equal); data curation (equal); formal analysis (equal); funding acquisition (equal); investigation (equal); methodology (equal); project administration (equal); resources (equal); software (equal); supervision (equal); visualization (equal); writing – original draft (equal); writing – review and editing (equal). **Vinay Prasad:** Conceptualization (equal); data curation (equal); formal analysis (equal); funding acquisition (equal); investigation (equal); methodology (equal); project administration (equal); resources (equal); software (equal); supervision (equal); validation (equal); visualization (equal); writing – original draft (equal); writing – review and editing (equal). **Matt Vassar:** Conceptualization (equal); data curation (equal); formal analysis (equal); funding acquisition (equal); investigation (equal); methodology (equal); project administration (equal); resources (equal); software (equal); supervision (equal); validation (equal); visualization (equal); writing – original draft (equal); writing – review and editing (equal).

## FUNDING INFORMATION

None.

## CONFLICT OF INTEREST STATEMENT

VP reports research funding from Arnold Ventures; royalties from Johns Hopkins Press, Medscape, and MedPage; honoraria from GrandRounds/lectures from universities, medical centers, nonprofits, professional societies, YouTube, and Substack; consulting from UnitedHealthcare and OptumRX; speaking fees from Evicore. Plenary Session podcast has Patreon backers. MV reports receipt of funding from the National Institute on Drug Abuse, the National Institute on Alcohol Abuse and Alcoholism, the US Office of Research Integrity, Oklahoma Center for Advancement of Science and Technology, and internal grants from Oklahoma State University Center for Health Sciences—all outside of the present work. All other authors have nothing to report.

## Data Availability

The data described in this article are openly available in the Open Science Framework at https://osf.io/vdr68/?view_only=78fb8b6f2e3e440aa71423b40ec8dd46.
